# Higher remnant cholesterol is associated with an increased risk of amnestic mild cognitive impairment: a community-based cross-sectional study

**DOI:** 10.3389/fnagi.2024.1332767

**Published:** 2024-02-12

**Authors:** Yating Ai, Chunyi Zhou, Ming Wang, Chongming Yang, Shi Zhou, Xinxiu Dong, Niansi Ye, Yucan Li, Ling Wang, Hairong Ren, Xiaolian Gao, Man Xu, Hui Hu, Yuncui Wang

**Affiliations:** ^1^School of Nursing, Hubei University of Chinese Medicine, Wuhan, China; ^2^Hubei Shizhen Laboratory, Hubei University of Chinese Medicine, Wuhan, China; ^3^Engineering Research Center of TCM Protection Technology and New Product Development for the Elderly Brain Health, Ministry of Education, Hubei University of Chinese Medicine, Wuhan, China; ^4^Tianyou Hospital, Wuhan University of Science and Technology, Wuhan, China; ^5^Research Support Center, Brigham Young University, Provo, UT, United States

**Keywords:** remnant cholesterol, amnestic mild cognitive impairment, Alzheimer’s disease, cross-sectional, older adults, community

## Abstract

**Background and aims:**

Amnestic mild cognitive impairment (aMCI) is the most common subtype of MCI, which carries a significantly high risk of transitioning to Alzheimer’s disease. Recently, increasing attention has been given to remnant cholesterol (RC), a non-traditional and previously overlooked risk factor. The aim of this study was to explore the association between plasma RC levels and aMCI.

**Methods:**

Data were obtained from Brain Health Cognitive Management Team in Wuhan (https://hbtcm.66nao.com/admin/). A total of 1,007 community-dwelling elders were recruited for this project. Based on ten tools including general demographic data, cognitive screening and some exclusion scales, these participants were divided into the aMCI (*n* = 401) and normal cognitive groups (*n* = 606). Physical examinations were conducted on all participants, with clinical indicators such as blood pressure, blood sugar, and blood lipids collected.

**Results:**

The aMCI group had significantly higher RC levels compared to the normal cognitive group (0.64 ± 0.431 vs. 0.52 ± 0.447 mmol/L, *p* < 0.05). Binary logistics regression revealed that occupation (*P*<0.001, OR = 0.533, 95%CI: 0.423–0.673) and RC (*p* = 0.014, OR = 1.477, 95% CI:1.081–2.018) were associated factors for aMCI. Partial correlation analysis, after controlling for occupation, showed a significant negative correlation between RC levels and MoCA scores (*r* = 0.059, *p* = 0.046), as well as Naming scores (*r* = 0.070, *p* = 0.026). ROC curve analysis demonstrated that RC levels had an independent predictive efficacy in predicting aMCI (AUC = 0.580, 95%CI: 0.544 ~ 0.615, *P* < 0.001).

**Conclusion:**

Higher RC levels were identified as an independent indicator for aMCI, particularly in the naming cognitive domain among older individuals. Further longitudinal studies are necessary to validate the predictive efficacy of RC.

## 1 Introduction

Mild cognitive impairment (MCI) is a transitional state between normal aging and dementia (Petersen et al., 2001; Langa and Levine, 2014). The key distinction between MCI and dementia is that the level of cognitive decline in MCI is not severe enough to significantly impact one’s daily functioning (Petersen et al., 2018). Amnestic mild cognitive impairment (aMCI) is the most common subtype of MCI that carries a high risk for transitioning into Alzheimer’s disease (AD), which unfortunately is irreversible and currently lacks effective treatment methods (Petersen, 2000; Van der Mussele et al., 2014). However, the aMCI stage still offers controllability and potential reversibility, which implies that the cognitive function of individuals with aMCI can be restored to a normal state or maintained relatively stable for a period of four to 5 years, or potentially even longer (US Preventive Services Task Force et al., 2020). This window of opportunity makes it the optimal period to delay or prevent the onset of AD. Our previous research discovered that aMCI often goes unnoticed in the communities; and Chinese older adults of aMCI and their families are less likely to actively seek medical attention until the condition has progressed to the dementia stage (Sun et al., 2018; Yang et al., 2021). Therefore, early identification of factors associated with aMCI becomes crucial.

Unfortunately, many studies primarily focus on exploring factors related to the progression from aMCI to AD (Van Rossum et al., 2012; Li et al., 2016; Rossini et al., 2022), rather than identifying risk factors specific to MCI itself. Some studies investigated associated factors of MCI, but solely relied on assessments such as the Mini-Mental State Examination (MMSE) or the Montreal Cognitive Assessment (MoCA), which may impact the reliability of research findings (Li et al., 2021; Huang et al., 2022; Zhong et al., 2023). Furthermore, the specific subtypes of MCI are often overlooked, leading to inconsistencies in research results.

Various factors may be associated with MCI, such as age, gender, education level, genetic factors, chronic disease factors, and lifestyle factors (Katayama et al., 2020; US Preventive Services Task Force et al., 2020; Huang et al., 2022; Zhong et al., 2023). Chronic disease factors such as hypertension, diabetes, hypercholesterolemia, heart disease, pulmonary disease (Katayama et al., 2020; US Preventive Services Task Force et al., 2020), osteoarthritis (Ribeiro et al., 2022), kidney disease (Viggiano et al., 2020) may affect cognitive function of the older individuals. Hobbies such as watching TV, reading, taking physical exercise, walking, playing cards/chess (Rundek and Bennett, 2006; Geda et al., 2011; Zhao et al., 2015), keeping pets (Friedmann et al., 2023), dancing (Zhu et al., 2020) may affect the advanced and instrumental daily living abilities of older individuals, as well as overall cognitive and executive functions. However, there is still no consensus on many of these factors. In a rigorous research design, chronic diseases and hobbies should be considered as control variables when exploring associated factors of aMCI.

Similarly, research on laboratory biological indicators of MCI has been inconclusive. Some studies have examined the relationship between individual indicators in peripheral blood and MCI, such as homocysteine (Bhargava et al., 2018), neutrophil lymphocyte ratio (An et al., 2019), standard deviation of red blood cell distribution width (Du et al., 2020), and fasting blood glucose (Li et al., 2021). Currently, there is a lack of specific biological diagnostic indicators, and thus it may be promising to further explore the predictive value of blood indicators for aMCI in older adults.

Hyperlipidemia was found to be a potential risk factor for cognitive impairment (Anstey et al., 2017). However, the association between plasma lipids and MCI among older individuals remains controversial (He et al., 2016). Cross-sectional or epidemiological studies were advocated to further investigate the role of blood lipids in MCI (McFarlane et al., 2020). This study diverted our effort to the relationship between remnant cholesterol (RC) and MCI, as it has been often overlooked or not fully explored (Sandesara et al., 2019).

RC refers to the cholesterol content present in remnants, which can be measured in a laboratory setting or calculated based on the values of LDL-C and HDL-C. These remnants are a subset of lipoproteins that are rich in triglycerides. RC particles are larger and more abundant, posing a greater risk on arterial endothelium (Packard, 2022). Evidence suggested that the relationship between RC and cognitive function can be attributed to the increased risk of cardiovascular disease secondary to higher RC levels (Xiao et al., 2023). This link is thought to be mediated by adverse effects on the arterial endothelium.

Research on the relationship between RC and cognitive function has been limited. A small sample (*n* = 36) cross-sectional study has preliminarily confirmed the correlation between RC and MCI (Zhang et al., 2023). Another study suggests that levels of RC are linked to verbal learning and memory function, suggesting that reducing RC levels could have potential benefits in preventing cognitive impairment in older individuals (Xie et al., 2022). Furthermore, accumulating evidence suggests that higher RC levels can increase the risk of residual atherosclerotic cardiovascular diseases (Wadström et al., 2022), stroke (Yang et al., 2023), hypertension (Chen et al., 2022), and diabetes mellitus (Petersen, 2004). Considering the extensive connection between these diseases and MCI, we hypothesized that plasma RC levels were associated with aMCI. Further researches are needed to validate the roles of RC in cognitive function.

To address these gaps, we conducted a community-based cross-sectional study, focusing on older individuals aged 65 and above. Through rigorous aMCI diagnosis, controlled for confounding factors, aimed to further investigate the relationship between RC levels and aMCI, and provide new data to support the exploration of the predictive value of RC in diagnosing aMCI.

## 2 Materials and methods

### 2.1 Participants

A multi-stage whole-group sampling was carried out from January 2022 to July 2022 to select older individuals from the communities under the jurisdiction of Wunancun Community Health Service Station in Wuchang District and Hongshan District Hospital of TCM, Wuhan City, Hubei Province, China. Participants were included and excluded by the criteria defined below.

#### 2.1.1 Inclusion criteria

① age ≥ 65 years old; ② having lived in the target communities in Wuhan for more than 1 year and not planning to move out within 2 years; ③ having sufficient visual and auditory discrimination to undergo neuropsychological testing; ④ provided informed consent for voluntary participation.

#### 2.1.2 Exclusion criteria

① those who did not meet the above criteria and had incomplete information; ② those who had cognitive impairment caused by other diseases like brain injury, drug poisoning, etc.; ③ those who had slurred conscious speech, psychiatric disorders, or severe heart, liver, or kidney diseases; ④ those who did not want to accept the study or could not cooperate for other reasons.

**Figure 1:**
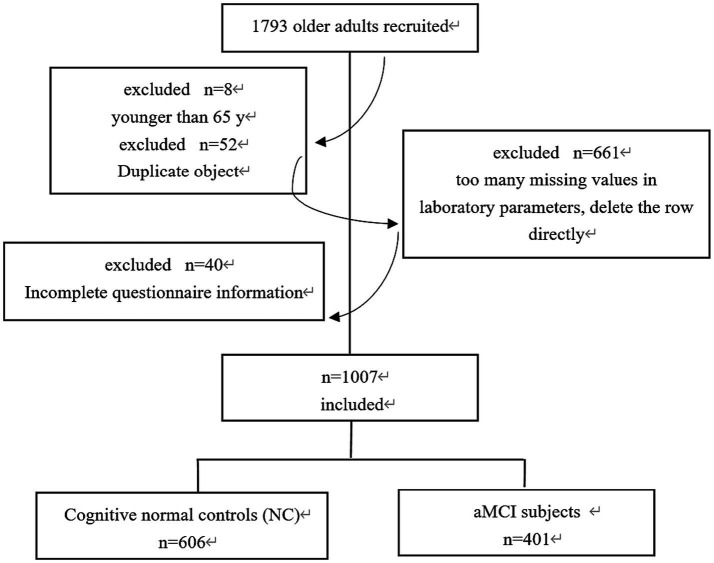
Flow chart detailing the derivation of the study sample.

As shown in Figure 1, the final sample of 1,007 participants were screened out for the analysis. All these subjects were examined by experienced geriatric psychiatrists according to the diagnostic criteria of cognitive impairment. They were divided into two groups: the normal cognitive group (NC) (*n* = 606) and the aMCI group (*n* = 401).

### 2.2 Cognitive evaluation

A total of 1793 older people participated in the questionnaire survey through the testing port of a brain benefiting cloud platform^1^ developed by the Brain Health Cognitive Management Team, Hubei University of Chinese Medicine. This cloud platform was developed based on two national projects, integrating cognitive function screening, intervention, follow-up and management of older adults in Wuhan communities. The assessment included general demographic data, cognitive screening, and some exclusion scales, taking approximately 30–50 min.

### 2.3 Diagnostic criteria for aMCI

According to the Petersen revised criteria (Writing Group of Dementia and Cognitive Impairment of Neurology Branch of Chinese Medical Association, 2010), the Chinese Guidelines for the Diagnosis and Treatment of Dementia and Cognitive Impairment (Chinese Society of Dementia and Cognitive Impairment, 2022), expert consensus (Shi et al., 2012), and the Chinese Guidelines for the Treatment of Dementia (Jinzhou et al., 2021; Hu et al., 2022) regarding the diagnostic criteria of aMCI include: ① complaints of memory impairment, confirmed by others; ② evidence of mild cognitive impairment or psycho-behavioral assessment confirmed by objective assessment; ③ MMSE score between 24 and 30 points, MoCA score less than 26 points; ④ intact or very slightly impaired activities of daily living, such as activities of daily living (ADL) score less than 16; ⑤ Clinical Dementia Rating Scale (CDR) = 0.5; ⑥ Hachinski Inchemic Score (HIS) <4; ⑧ Hamilton Depression Scale (HAMD) <20.

### 2.4 Blood sampling and laboratory tests

After fasting overnight for 12 h, blood samples were collected from the anterior cubital vein by professionals from 7:30 am to 9:00 am, and a coded container was used to collect clean morning urine samples as required. Laboratory indicators including fasting blood glucose, Total cholesterol (TC), triglycerides (TG), high-density lipoprotein cholesterol (HDL-C) and low-density lipoprotein cholesterol (LDL-C) were tested by the hospital laboratory center and uploaded to the hospital laboratory information system. The RC was calculated by subtracting HDL-C and LDL-C from total cholesterol (TC): RC = TC – HDL-C − LDL-C.

### 2.5 Ethical statement

This study followed the guidelines of the World Medical Association Declaration of Helsinki. The Medical Ethics Committee of Hubei University of Chinese Medicine reviewed and approved the study protocol (No.: 2019IEC003). All participants signed informed consent for the study. Participants’ personal information was strictly confidential, and the medical examination number was used as the unique identification code instead of their names.

### 2.6 Statistical analysis

The data were exported to Excel format from the background management port of the brain benefiting cloud platform.^2^ Statistical analysis was performed using SPSS 25.0 and Python 3.9. Differences in the demographics and laboratory tests between the two groups were first examined with statistical tests appropriate for the variable types. Specifically, continuous data were expressed as mean and standard deviation (mean ± SD), normal tests, homogeneity of variance tests and independent sample t-tests were used for continuous variables; Categorical variables were expressed as frequencies (%) and the chi-square tests were chosen when compare the two groups (NC vs. aMCI). Second, binary logistic regression analysis was run to determine the associated factors of aMCI, and the results were reported in odds ratio (OR) and 95% confidence intervals (CI). Third, partial correlation analysis was conducted to examine the correlation between RC and cognitive function in aMCI elders. A two-sided *P*<0.05 was considered statistically significant. As the last step, receiver operating characteristic (ROC) curve analysis was conducted to explore the predictive efficacy of RC in predicting aMCI, irrespective of other covariates.

## 3 Results

### 3.1 Demographic and clinical features of the NC and aMCI groups

To minimize confounding factors and ensure the comparability of the research participants, dementia family history, chronic disease history and hobbies of older individuals were controlled (Supplementary Tables S1, S2).

Table 1 shows that compared to the NC group, the aMCI group had a higher proportion of manual work and lower income levels (*p* < 0.05); Besides, the aMCI group also performed worse in cognitive subtests except orientation (all *p* < 0.05).

**Table 1:** Demographic properties and laboratory parameters of the two groups (*n* = 1,007).

variables	NC(n = 606)	aMCI(n = 401)	χ^2^ or t	*P*
Age, years	71.14 ± 4.944	71.25 ± 4.708	−0.342	0.733
Gender, male *n* (%)	255 (42.1)	153 (38.2)	1.542	0.214
Education, *n* (%)			6.029	0.110
Primary school and below	93 (15.3)	61 (15.2)		
Junior high school	187 (30.9)	149 (37.2)		
Technical secondary school/high school	198 (32.7)	126 (31.4)		
College degree or above	128 (21.1)	65 (16.2)		
Marriage, single^#^ (%)	122 (20.1)	75 (18.7)	0.313	0.576
Living, single (%)	62 (10.2)	37 (9.2)	0.274	0.600
Occupation^^^, *n* (%)			54.105	<0.001^*^
Manual	332 (54.8)	310 (77.3)		
Mental	221 (36.5)	68 (17.0)		
Uncertain	53 (8.7)	23 (5.7)		
Income, over ¥4,000(%)	229 (37.8)	121 (30.2)	6.170	0.013^*^
Family history, yes (%)	51 (8.4)	23 (5.7)	2.546	0.111
Hypertension, yes (%)	282 (46.5)	178 (44.4)	0.448	0.503
Diabetes, yes (%)	115 (19.0)	82 (20.4)	0.332	0.564
Hyperlipidemia, yes (%)	63 (10.4)	50 (12.5)	1.041	0.308
SBP (mmHg)	134.23 ± 19.921	133.34 ± 18.975	0.705	0.481
DBP (mmHg)	75.68 ± 11.638	76.82 ± 11.043	−1.541	0.124
FBGLU (mmol/L)	5.70 ± 1.485	5.63 ± 1.573	0.698	0.485
TC (mmol/L)	4.86 ± 1.030	4.92 ± 1.107	−0.830	0.407
Triglyceride (mmol/L)	1.61 ± 1.217	1.58 ± 1.069	0.416	0.677
HDL-C (mmol/L)	1.45 ± 0.435	1.38 ± 0.348	2.809	0.005^*^
LDL-C (mmol/L)	2.89 ± 0.822	2.89 ± 0.851	−0.101	0.919
HDL-C/ LDL-C (mmol/L)	0.55 ± 0.266	0.52 ± 0.201	2.307	0.021^*^
RC (mmol/L)	0.52 ± 0.447	0.64 ± 0.431	−4.293	<0.001^*^
MMSE	26.69 ± 3.074	27.19 ± 2.008	−3.105	0.002^*^
MoCA	22.18 ± 5.077	21.07 ± 3.367	4.159	<0.001^*^
Visuospatial and Executive	3.28 ± 1.445	3.04 ± 1.220	2.786	0.005^*^
Naming	2.41 ± 0.814	2.29 ± 0.794	2.455	0.014^*^
Attention and Calculation	5.74 ± 1.346	5.91 ± 0.998	−2.333	0.020^*^
Language	1.63 ± 0.840	1.45 ± 0.740	3.724	<0.001^*^
Abstraction	1.14 ± 0.758	0.96 ± 0.690	3.773	<0.001^*^
Recall	2.33 ± 1.604	1.71 ± 1.329	6.668	<0.001^*^
Orientation to time and place	5.66 ± 0.733	5.72 ± 0.622	−1.524	0.128

P.S.: ^#^means unmarried/widowed/divorced; ^^^means occupation one’s engaged before retirement; ^*^means *P* < 0.05; FBGLU, Fasting blood glucose; TC, Total cholesterol; RC, Remnant cholesterol.

### 3.2 Lipid parameters of the NC and aMCI groups

Compared to the NC group, the aMCI group had lower HDL-C, HDL-C/LDL-C levels, and higher RC levels (*p* < 0.05); However, there were no significant differences in TC, TG, and LDL-C levels between the two groups (*P* > 0.05), more details see Table 1.

### 3.3 Logistic regression analysis of the possible correlates for aMCI

Table 2 shows the results of binary logistics regression with aMCI as a dependent variable (0 = No, 1 = Yes); Model 1 did not control any covariates, RC was treated as the independent variables, and the results showed that RC increased the risk of aMCI (*p* = 0.000, OR = 1.890, 95%CI: 1.396–2.558). Taking into account statistically significant variables (*p* < 0.05), Model 2 further controlled for occupation, income, HDL-C and HDL-C/LDL-C, the relationship between RC and aMCI has been weakened but still exists (*p* = 0.046, OR = 1.453, 95%CI: 1.006–2.097), other associated factors of aMCI were occupation (*P* < 0.001, OR = 0.543, 95%CI: 0.430–0.685) and income (*p* = 0.018, OR = 0.715, 95%CI: 0.542–0.943). Taking into account professionally significant variables, excluding multicollinear covariates, and simplifying the model, Model 3 retained significant variables in univariate analysis, and further controlled for age, gender and education, excluded HDL and HDL-C/ LDL-C, the results did not change. Model 3 showed that occupation (*P* < 0.001, OR = 0.533, 95%CI: 0.423–0.673) and RC (*p* = 0.014, OR = 1.477, 95%CI: 1.081–2.018) were associated factors of aMCI. Specifically, the risk of aMCI was higher in elders engaged in manual work before retirement compared to those engaged in mental work. RC levels were positively associated with the possibility of aMCI, in that every 1-unit (mmol/L) increase in RC, the odds of aMCI increased by 1.477 times (95%CI: 1.081–2.018).

**Table 2:** Results of logistic regression analysis of the possible correlates for aMCI.

Variable	*β*	SE	Wals	*P*	OR (95%CI)
Model 1					
RC	0.636	0.155	16.968	0.000	1.890(1.396 ~ 2.558)
constant	−0.780	0.111	49.520	0.000	0.458
Model 2					
occupation	−0.611	0.119	26.435	0.000	0.543(0.430 ~ 0.685)
income	−0.335	0.141	5.637	0.018	0.715(0.542 ~ 0.943)
HDL-C	−0.129	0.220	0.343	0.558	0.879(0.571 ~ 1.353)
HDL-C/ LDL-C	0.123	0.386	0.102	0.749	1.131(0.531 ~ 2.412)
RC	0.373	0.187	3.976	0.046	1.453(1.006 ~ 2.097)
constant	0.793	0.432	3.370	0.066	2.210
Model 3					
age	−0.001	0.014	0.007	0.934	0.999(0.972 ~ 1.026)
gender	−0.051	0.149	0.117	0.733	0.950(0.710 ~ 1.273)
education	−0.069	0.071	0.929	0.335	0.934(0.812 ~ 1.074)
occupation	−0.628	0.119	27.973	0.000	0.533(0.423 ~ 0.673)
income	−0.284	0.166	2.938	0.087	0.753(0.544 ~ 1.042)
RC	0.390	0.159	6.010	0.014	1.477(1.081 ~ 2.018)
constant	1.026	1.118	0.843	0.359	2.791

### 3.4 Associations between RC and cognitive performance in aMCI elders

Partial correlation analysis was conducted to control for occupation. Table 3 showed a significant negative correlation between RC levels and MoCA score (*r* = 0.059, *p* = 0.046), Naming score (*r* = 0.070, *p* = 0.026), indicating that higher RC levels were associated with worse cognitive performance in aMCI participants. No significant correlation with other subtest was found (*p* > 0.05).

**Table 3:** Correlations between RC levels and cognitive performance.

		MOCA	Visuospatial and executive	Naming	Attention and calculation	Language	Abstraction	Recall	Orientation
RC	*r*	−0.063	−0.059	−0.070	−0.008	−0.022	−0.051	−0.034	−0.044
	*P*	0.046^*^	0.060	0.026^*^	0.793	0.491	0.107	0.287	0.165

P.S.: control variable: occupation. *P < 0.05, considered statistically significant.

### 3.5 The independent predictive efficacy of RC level on aMCI

**Figure 2:**
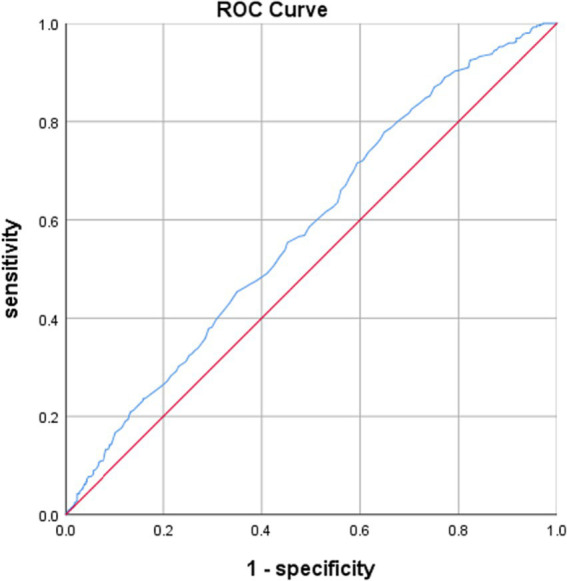
ROC curves for the predictive efficacy of RC on aMCI.

ROC curve analysis indicated that RC levels had an independent predictive efficacy in predicting aMCI, with an area under the curve (AUC) of 0.580 (*p* < 0.001, 95%CI: 0.544–0.615), see Table 4. The results suggest that higher RC levels were associated with a higher likelihood of aMCI, see Figure 2.

**Table 4:** AUC of lipid parameters to predict aMCI.

variable	AUC	SE	*P*	95% CI	
				Lower	Upper
RC	0.580	0.018	0.000	0.544	0.615
TC	0.513	0.019	0.487	0.476	0.550
TG	0.493	0.019	0.689	0.456	0.529
HDL-C	0.459	0.018	0.028	0.423	0.495
LDL-C	0.503	0.019	0.890	0.466	0.539
HL	0.477	0.019	0.224	0.441	0.514

Independent predictive value of other blood lipid parameters for aMCI were shown in Table 4.

## 4 Discussions

This study investigated the correlations between blood lipids and aMCI and subsequently discovered that RC levels were associated with aMCI. To our knowledge, this is the first study to establish this connection using data from over 1,000 community older individuals. Specifically, the key findings include: ① the aMCI group exhibited higher RC levels compared to the group with normal cognitive function; ② plasma RC levels were positively linked to the likelihood of developing aMCI; ③ RC levels were associated with impaired cognitive performance, particularly in the naming cognitive domain, among older individuals with aMCI; ④ plasma RC levels independently predicted aMCI.

These results were consistent with subgroup analysis result that the association between RC and the risk of cardiovascular events is not dependent on factors such as pre-existing diseases, diabetes, total cholesterol, triglyceride levels, ApoB (apolipoprotein B) levels, or BMI stratification (Yang et al., 2023). As the regression modeling controlled for comorbidities such as hypertension, diabetes, or stroke, chronic diseases, and occupations, no concerns seemed to be necessary regarding whether the risk of aMCI associated with RC depends on comorbidities and occupation.

Is it possible that the risk of RC was dependent on other lipid parameters? To address this question, a collinearity diagnosis was performed on blood lipid parameters, and no multicollinearity was found among the blood indicators related to cognitive function (tolerance: 0.248–0.564, VIF:1.774–4.036). No additional differences were found in TG, TC and LDL-C between aMCI elders and normal controls. However, the average HDL-C level and HDL/LDL ratio in the aMCI group were lower, compared to the normal group. A cross-sectional study reported that higher circulating TC, HDL-C and HDL/LDL ratio indicated an increased risk of MCI (Guo et al., 2020). On the other hand, another observational study including 125,727 individuals suggested that higher plasma triglycerides concentrations were associated with an increased risk of non-Alzheimer dementia and ischemic stroke but not with AD and aMCI (Nordestgaard et al., 2021). Therefore, the association between blood lipid parameters and cognitive function remains controversial.

The biological mechanisms of the association between RC and aMCI are beyond the scope of this study and still unclear. Several possibilities can be considered, with the first involving genetic factors. Using Mendelian randomization, a previous research found that APOE4 carriers had less favorable lipid profiles, which were associated with a greater risk of dementia and cognitive impairment (Dunk et al., 2023). Second, RC may interact with beta-amyloid (Aβ), which is biologically plausible (Zhang et al., 2023). Cholesterol transport may be involved in the development and progression of cognitive impairment (Zhang et al., 2023). Last, the association between high RC levels and aMCI could be explained by the pathophysiological processes of atherosclerosis (Hu et al., 2022; Nordestgaard et al., 2022). Other potential mechanisms including anti-oxidation, anti-inflammation, anti-thrombosis, and modulation of immune function (Yang et al., 2023). All these possible explanations entail further studies.

The demographic characteristics were not found statistically different in age, gender and education attainment between aMCI group and the normal controls. However, the aMCI group had a higher average age, a larger proportion of women, and a lower proportion of individuals with high school education or above, which is consistent with previous research (Garibotto et al., 2008; Overton et al., 2019; US Preventive Services Task Force et al., 2020; Huang et al., 2022). Additionally, compared to the normal group, the aMCI group had a lower average monthly income, which aligns with previous findings (Guo et al., 2023). These demographics differences did not shadow the unique association of RC with aMCI identified from the logistic regression, as they were controlled.

### 4.1 Limitations and strengthens

There are some limitations to this study. First, it was only a cross-sectional study, and RC levels were measured only once. Although RC may change over time, it is generally considered relatively stable, so this limitation would not significantly impact the main aim of the study. However, in order to establish a causal relationship between RC and aMCI, more longitudinal or randomized controlled trials (RCT) studies are needed. Second, the study population consisted of individuals residing in an urban area of central China, which may limit the generalizability of the findings to rural populations and other ethnic groups; Third, the intermittent outbreaks of COVID-19 in Wuhan China caused missing laboratory indicators in many participants, whom had to be excluded from the analyses. It is uncertain whether this exclusion affected the representativeness of the sample and result.

Despite these limitations, this study still has several important strengths. This study design was rigorous, with cognitive function assessments conducted by experienced geriatric psychiatrists according to strict criteria. The diagnosis of MCI did not rely solely on one scale such as MMSE or MoCA, a complete diagnostic program enhanced the validity of the findings. Additionally, the detailed questionnaire allowed for the consideration of various important confounding factors related to the associations found. The study also excluded the effects of diseases and vascular factors on MCI and included older individuals with aMCI who mainly experienced memory decline, ensuring homogeneity within the study sample. Furthermore, our research team developed a cloud platform for data collection and storage, which minimized potential errors in data entry that could arise with paper questionnaires.

## 5 Conclusion

The study findings indicated that higher RC levels were identified as an independent indicator for aMCI, particularly in the naming cognitive domain among older individuals. In addition to conventional lipid parameters, clinicians should pay close attention to RC for prevention of MCI. Further longitudinal studies are necessary to validate the predictive efficacy of RC.

## Data availability statement

The raw data supporting the conclusions of this article will be made available by the authors, without undue reservation.

## Ethics statement

The studies involving humans were approved by the Medical Ethics Committee of Hubei University of Chinese Medicine. The studies were conducted in accordance with the local legislation and institutional requirements. The participants provided their written informed consent to participate in this study.

## Author contributions

YA: Conceptualization, Data curation, Formal analysis, Funding acquisition, Investigation, Methodology, Project administration, Software, Writing – original draft, Writing – review & editing. CZ: Conceptualization, Formal analysis, Investigation, Methodology, Software, Validation, Writing – original draft, Writing – review & editing. MW: Data curation, Formal analysis, Investigation, Resources, Validation, Writing – review & editing. CY: Writing – original draft, Writing – review & editing, Methodology. SZ: Conceptualization, Data curation, Formal analysis, Investigation, Writing – review & editing. XD: Conceptualization, Data curation, Formal analysis, Investigation, Writing – review & editing. NY: Conceptualization, Data curation, Formal analysis, Investigation, Writing – review & editing. YL: Data curation, Formal analysis, Investigation, Writing – review & editing. LW: Conceptualization, Methodology, Writing – review & editing. HR: Conceptualization, Formal analysis, Writing – review & editing. XG: Methodology, Software, Writing – review & editing. MX: Formal analysis, Methodology, Writing – review & editing. HH: Conceptualization, Data curation, Formal analysis, Funding acquisition, Methodology, Project administration, Resources, Supervision, Writing – original draft, Writing – review & editing. YW: Conceptualization, Data curation, Formal analysis, Methodology, Project administration, Supervision, Validation, Writing – original draft, Writing – review & editing.

## Footnotes

^1^https://hbtcm.66nao.com/assess/

^2^https://hbtcm.66nao.com/admin/
