# Supplementary material for: Higher remnant cholesterol is associated with an increased risk of amnestic mild cognitive impairment: a community-based cross-sectional study
Source: Front Aging Neurosci. 2024 Feb 12;16:1332767. doi: 10.3389/fnagi.2024.1332767 (PMC10894954; doi:10.3389/fnagi.2024.1332767)
Supplement: Supplementary file 1 [file Data_Sheet_1.docx]

Supplementary Material

Higher Remnant Cholesterol Associated with an Increased Risk of Amnestic Mild Cognitive Impairment? A Community-Based Cross-Sectional Study

**Yating Ai^1,2,3†^, Chunyi Zhou^1†^, Ming Wang^4^, Shi Zhou^1^, Xinxiu Dong^1^, Niansi Ye^1^, Yucan Li^1^, Ling Wang^1^, Hairong Ren^1^, Xiaolian Gao^1^, Man Xu^1^, Hui Hu^1,2,3*^, Yuncui Wang^1,2,3*^**

*** Correspondence:**

Hui Hu, PhD

School of Nursing

Hubei University of Chinese Medicine

Email: zhongyi90@163.com

Yuncui Wang, PhD

School of Nursing

Hubei University of Chinese Medicine

Email: yuncui_wang@hbtcm.edu.cn

# Supplementary Tables

**Supplementary Table 1.** dementia family history, chronic disease history of two groups.

|  | NC(n=606) | | aMCI(n=401) | | *χ^２^* | *P* |
| --- | --- | --- | --- | --- | --- | --- |
|  | n | % | n | % |  |  |
| dementia family history |  |  |  |  | 2.546 | 0.111 |
| Yes | 51 | 8.4% | 23 | 5.7% |  |  |
| No | 555 | 91.6% | 378 | 94.3% |  |  |
| hypertension |  |  |  |  | 0.448 | 0.503 |
| Yes |  |  |  |  |  |  |
| No | 324 | 53.5% | 223 | 55.6% |  |  |
| diabetes |  |  |  |  | 0.332 | 0.564 |
| Yes | 115 | 19.0% | 82 | 20.4% |  |  |
| No | 491 | 81.0% | 319 | 79.6% |  |  |
| hyperlipidemia |  |  |  |  | 1.041 | 0.308 |
| Yes | 63 | 10.4% | 50 | 12.5% |  |  |
| No | 543 | 89.6% | 351 | 87.5% |  |  |
| heart disease |  |  |  |  | 0.462 | 0.497 |
| Yes | 83 | 13.7% | 49 | 12.2% |  |  |
| No | 523 | 86.3% | 352 | 87.8% |  |  |
| mild liver/kidney dysfunction |  |  |  |  | 0.146 | 0.702 |
| Yes | 24 | 4.0% | 14 | 3.5% |  |  |
| No | 582 | 96.0% | 387 | 96.5% |  |  |
| stroke |  |  |  |  | 0.034 | 0.854 |
| Yes | 38 | 6.3% | 24 | 6.0% |  |  |
| No | 568 | 93.7% | 377 | 94.0% |  |  |
| osteoarthritis |  |  |  |  | 0.007 | 0.934 |
| Yes | 73 | 12.0% | 49 | 12.2% |  |  |
| No | 533 | 88.0% | 352 | 87.8% |  |  |
| chronic respiratory disease |  |  |  |  | 0.082 | 0.774 |
| Yes | 12 | 2.0% | 9 | 2.2% |  |  |
| No | 594 | 98.0% | 392 | 97.8% |  |  |
| chronic liver disease |  |  |  |  | 1.025 | 0.311 |
| Yes | 21 | 3.5% | 19 | 4.7% |  |  |
| No | 585 | 96.5% | 382 | 95.3% |  |  |
| metabolic diseases |  |  |  |  | 0.084 | 0.773 |
| Yes | 39 | 6.4% | 24 | 6.0% |  |  |
| No | 567 | 93.6% | 377 | 94.0% |  |  |
| mild visual/auditory impairment |  |  |  |  | 1.412 | 0.235 |
| Yes | 10 | 1.7% | 11 | 2.7% |  |  |
| No | 596 | 98.3% | 390 | 97.3% |  |  |
| chronic gastrointestinal diseases |  |  |  |  | 0.217 | 0.641 |
| Yes | 25 | 4.1% | 19 | 4.7% |  |  |
| No | 581 | 95.9% | 382 | 95.3% |  |  |
| number of chronic diseases |  |  |  |  | 3.691 | 0.297 |
| 0 | 132 | 21.8% | 88 | 21.9% |  |  |
| 1 | 246 | 40.6% | 175 | 43.6% |  |  |
| 2 | 151 | 24.9% | 80 | 20.0% |  |  |
| 3 | 77 | 12.7% | 58 | 14.5% |  |  |

**Supplementary Table 2.** personal hobbies of two groups.

|  | NC(n=606) | | MCI(n=401) | | *χ^２^* | *P* |
| --- | --- | --- | --- | --- | --- | --- |
|  | n | % | n | % |  |  |
| watch TV |  |  |  |  | 0.004 | 0.949 |
| Yes | 240 | 39.6% | 158 | 39.4% |  |  |
| No | 366 | 60.4% | 243 | 60.6% |  |  |
| walking |  |  |  |  | 0.001 | 0.975 |
| Yes | 285 | 47.0% | 189 | 47.1% |  |  |
| No | 321 | 53.0% | 212 | 52.9% |  |  |
| listen to radio/music |  |  |  |  | 0.447 | 0.504 |
| Yes | 14 | 2.3% | 12 | 3.0% |  |  |
| No | 592 | 97.7% | 389 | 97.0% |  |  |
| play cards |  |  |  |  | 0.851 | 0.356 |
| Yes | 75 | 12.4% | 42 | 10.5% |  |  |
| No | 531 | 87.6% | 359 | 89.5% |  |  |
| Planting flowers/keeping pets |  |  |  |  | 3.010 | 0.083 |
| Yes | 30 | 5.0% | 11 | 2.7% |  |  |
| No | 576 | 95.0% | 390 | 97.3% |  |  |
| reading |  |  |  |  | 1.094 | 0.296 |
| Yes | 53 | 8.7% | 43 | 10.7% |  |  |
| No | 553 | 91.3% | 358 | 89.3% |  |  |
| fishing |  |  |  |  | 3.211 | 0.073 |
| Yes | 18 | 3.0% | 5 | 1.2% |  |  |
| No | 588 | 97.0% | 396 | 98.8% |  |  |
| physical exercise |  |  |  |  | 0.487 | 0.485 |
| Yes | 119 | 19.6% | 86 | 21.4% |  |  |
| No | 487 | 80.4% | 315 | 78.6% |  |  |
| group travel |  |  |  |  | 0.605 | 0.437 |
| Yes | 16 | 2.6% | 14 | 3.5% |  |  |
| No | 590 | 97.4% | 387 | 96.5% |  |  |
| community activities |  |  |  |  | 0.169 | 0.681 |
| Yes | 24 | 4.0% | 18 | 4.5% |  |  |
| No | 582 | 96.0% | 383 | 95.5% |  |  |
| play chess |  |  |  |  | 0.187 | 0.666 |
| Yes | 14 | 2.3% | 11 | 2.7% |  |  |
| No | 592 | 97.7% | 390 | 97.3% |  |  |
| dancing |  |  |  |  | 0.007 | 0.933 |
| Yes | 34 | 5.6% | 23 | 5.7% |  |  |
| No | 572 | 94.4% | 378 | 94.3% |  |  |
| singing |  |  |  |  | 2.564 | 0.109 |
| Yes | 28 | 4.6% | 28 | 7.0% |  |  |
| No | 578 | 95.4% | 373 | 93.0% |  |  |
| others (physical) |  |  |  |  | 1.491 | 0.222 |
| Yes | 31 | 5.1% | 14 | 3.5% |  |  |
| No | 575 | 94.9% | 387 | 96.5% |  |  |
| others (mental) |  |  |  |  | 0.054 | 0.815 |
| Yes | 26 | 4.3% | 16 | 4.0% |  |  |
| No | 580 | 95.7% | 385 | 96.0% |  |  |
| using electronic devices |  |  |  |  | 0.678 | 0.410 |
| Yes | 461 | 76.1% | 314 | 78.3% |  |  |
| No | 145 | 23.9% | 87 | 21.7% |  |  |
| number of hobbies |  |  |  |  | 3.511 | 0.173 |
| 1 or less | 286 | 47.2% | 193 | 48.1% |  |  |
| 2 | 242 | 39.9% | 142 | 35.4% |  |  |
| 3 or more | 78 | 12.9% | 66 | 16.5% |  |  |
| whether to persist in hobbies |  |  |  |  | 3.233 | 0.357 |
| as usual | 500 | 82.5% | 337 | 84.0% |  |  |
| mild disorder | 53 | 8.7% | 34 | 8.5% |  |  |
| abandoning complex hobbies | 33 | 5.4% | 13 | 3.2% |  |  |
| keep only simple foreign affairs | 20 | 3.3% | 17 | 4.2% |  |  |
